# Impact of training and structured medication review on medication appropriateness and patient-related outcomes in nursing homes: results from the interventional study InTherAKT

**DOI:** 10.1186/s12877-019-1263-3

**Published:** 2019-09-18

**Authors:** Angelika Mahlknecht, Laura Krisch, Nadja Nestler, Ulrike Bauer, Nina Letz, Daniel Zenz, Jochen Schuler, Laura Fährmann, Georg Hempel, Maria Flamm, Jürgen Osterbrink

**Affiliations:** 1 Institute of General Practice, Family Medicine and Preventive Medicine, Paracelsus Medical University, Strubergasse 21, 5020 Salzburg, Austria; 2 Institute of Nursing Science and Practice, Paracelsus Medical University, Strubergasse 21, 5020 Salzburg, Austria; 3 smart-Q Softwaresysteme GmbH, BioMedizinZentrum Bochum, Universitätsstraße 136, 44799 Bochum, Germany; 4 Department of Pharmaceutical and Medical Chemistry - Clinical Pharmacy, Westfaelische Wilhelms-University, Corrensstraße 48, 48149 Muenster, Germany

**Keywords:** Drug safety, Appropriateness review, Nursing homes, Interprofessional relations, Health information technology

## Abstract

**Background:**

Uncoordinated interprofessional communication in nursing homes increases the risk of polypharmacy and inappropriate medication use. This may lead to augmented frequency of adverse drug events, hospitalizations and mortality. The aims of this study were (1) to improve interprofessional communication and medication safety using a combined intervention and thus, (2) to improve medication appropriateness and health-related outcomes of the included residents.

**Methods:**

The single-arm interventional study (2014–2017) was conducted in Muenster, Germany and involved healthcare professionals and residents of nursing homes.

The intervention consisted of systematic education of participating healthcare professionals and of a structured interprofessional medication review which was performed via an online communication platform.

The primary endpoint was assessed using the Medication Appropriateness Index MAI. Secondary endpoints were: cognitive performance, delirium, agitation, mobility, number of drugs, number of severe drug-drug interactions and appropriateness of analgesics.

Outcomes were measured before, during and after the intervention. Data were analyzed using descriptive and inference-statistical methods.

**Results:**

Fourteen general practitioners, 11 pharmacists, 9 nursing homes and 120 residents (*n* = 83 at all testing times) participated.

Overall MAI sum-score decreased significantly over time (mean reduction: -7.1, CI_95%_ -11.4 – − 2.8; median = − 3.0; d_Cohen_ = 0.39), especially in cases with baseline sum-score ≥ 24 points (mean reduction: -17.4, CI_95%_ -27.6 – − 7.2; median = − 15.0; d_Cohen_ = 0.86).

MAI sum-score of analgesics also decreased (d_Cohen_ = 0.45). Mean number of severe drug-drug interactions rose slightly over time (d_Cohen_ = 0.17). The proportion of residents showing agitated behavior diminished from 83.9 to 67.8%. Remaining secondary outcomes were without substantial change.

**Conclusion:**

Medication appropriateness increased particularly in residents with high baseline MAI sum-scores. Cognitive decline of participating residents was seemingly decelerated when compared with epidemiologic studies. A controlled trial is required to confirm these effects. Interprofessional interaction was structured and performance of medication reviews was facilitated as the online communication platform provided unlimited and consistent access to all relevant and updated information.

**Trial registration:**

DRKS Data Management, ID: DRKS00007900, date of registration: 2015-09-02 (retrospectively registered i.e. 6 weeks after commencement of the first data collection).

## Background

Polypharmacy and inappropriate medication use are major health concerns and represent common phenomena in older persons [1], leading to adverse drug events (ADEs) [2], increased hospitalization rates [3, 4] and mortality [5]. Residents of nursing homes (NHs) are at a particularly high risk of polypharmacy and inappropriate medication [5] due to complex and multiple comorbidities.

In Germany, most drug prescriptions for NH residents (NHRs) are performed by general practitioners (GPs), but also by other physicians [6] (e.g. specialists in neurology or psychiatry). Usually several different GPs and pharmacists working in offices or community pharmacies respectively supply one NH. Pharmacists dispense the medication, control drug storage and train the nurses in adequate delivery of drugs. They also perform analyses of drug-drug interactions (DDIs) but are not consulted regularly for complete medication reviews. Frequencies of NH visits performed by GPs, other physicians or pharmacists are variable and sometimes inconsistent. Nurses deliver medications and monitor the residents’ clinical condition [7].

The involvement of numerous different providers in the medication process leads to the demand for a regular exchange of information between all healthcare professionals concerned and for a periodic review and adjustment of the medication; however, previous studies have shown that this fails to occur regularly [8] which entails an increased risk of inappropriate prescribing [9]. Hence, a need for improved interprofessional communication is highlighted [10].

Health information technology (HIT) interventions are considered to be valuable in this regard, however, their impact on interprofessional collaboration is unclear [11]. A US controlled study applied prospective medication reviews performed by pharmacists, HIT-supported patient assessment, formalized pharmaceutical care planning in NHRs at high risk for medication-related problems, and direct communication with prescribers and found a positive effect on medication appropriateness, but no changes in number of medications, hospitalizations and mortality [12]. A current Belgian cluster-RCT with a multi-faceted, complex intervention in NHs applies a web tool for sharing patient-related information between healthcare professionals, preparing medication reviews and generating standardized reports of multi-disciplinary case conferences [13].

Nevertheless, up to now electronic tools have rarely been used as *direct* interprofessional communication instruments within the medication process in NHs. In general, previous HIT interventions aiming at improving medication safety frequently used computerized order entry and clinical decision support systems (CPOE/CDS) [14] by addressing various steps of the *prescription* process. Positive effects on quality of prescribing (improved guideline adherence, reduction of medication errors) and partly on surrogate outcomes (reduction of ADEs) were demonstrated [15, 16], but the use of these systems was limited in NHs [14, 17] and patient-related outcomes (e.g. mortality/hospitalizations) were studied only occasionally with inconsistent results [14, 18]. CPOE systems have also shown negative effects on communication, e.g. misconceptions about abrogated needs of face-to-face communication [19]. An American cluster-randomized controlled trial (RCT) used a HIT intervention targeting the *monitoring* stage of the medication process in NHs by using a software which evaluated medication regimens, identified patients at risk for falls and/or delirium and generated specific monitoring plans; the study found no effect on falls, but positive effects on delirium, hospitalization rates and mortality for newly admitted NHRs of the intervention group [20].

Other interventions addressing medication safety in NHs consisted of medication reviews, educational programs or multi-disciplinary case conferences and seemed to also be effective in reducing inappropriate prescribing [21], but couldn’t show consistent improvements of patient-related outcomes: some studies found a reduction of falls [22, 23], delirium [24] and hospital days [25], while no changes regarding mortality [10, 22, 25, 26], residents’ behavior [27], hospitalizations [22, 23, 26], quality of life [23], functional status or cognitive skills [22] were noted. Nevertheless, it seems plausible that enhanced interprofessional communication and consequent medication surveillance impacts patients’ outcomes favorably.

The aim of the InTherAKT study (“Initiative zur Therapiesicherheit in der Altenhilfe durch Kooperation und Teamwork”- Initiative for medication safety in long-term care via cooperation and teamwork) was to improve interprofessional collaboration within the medication process in NHs by a combined intervention consisting of systematic education of participating healthcare professionals and a structured interprofessional medication review via an online communication platform. The study evaluated changes in medication appropriateness as well as patient-related outcomes and changes in interprofessional communication.

### Study hypothesis

1. The intervention improves appropriateness of drug prescriptions for included NHRs (primary outcome).
2. The intervention has effects on patient-related outcomes (cognitive skills, mobility, agitation, delirium), number of drugs, severe DDIs and on appropriateness of analgesics (secondary outcomes).

The impact on interprofessional communication was investigated using a qualitative approach; these results will be reported in a separate publication.

## Methods

Details of methodology and study protocol have been published previously [7] and are summarized in the present article. The study adheres to CONSORT guidelines.

### Study design and population

The single-arm interventional study (2014–2017) involved healthcare professionals operating in NH care (GPs, nurses, pharmacists) and NHRs in Muenster, Germany.

### Recruitment and sample size

Recruitment (January 2015 – July 2015) was started with GPs as participation of all other healthcare professionals depended from GPs’ disposition to attend. GPs were invited by written information (newsletter of the GP association of Muenster to all its 120 members), an on-site information event and personal visits. Additionally, to achieve the aspired number of NHRs, physicians of the GP association of Muenster holding contracts for intensified treatment [7] of NHRs were addressed specifically. Participating GPs recruited their patients living in NHs and fulfilling the inclusion criteria. Corresponding NHs and pharmacists were recruited by the project team.

*Inclusion criteria (residents)*: written informed consent (resident/legal representative), age ≥ 65 years, ≥1 drug prescription/s.

*Exclusion criteria (residents)*: missing consent, insufficient cognitive performance and no legal representative, acute life-threatening situation, quarantine (isolation due to infectious diseases).

*Inclusion criteria (professional groups)*: GPs treating NHRs, pharmacists supplying participating NHs, nurses with ≥3 year state-approved training.

*Power calculation* was performed with a power of 0.8 (1-β = 80%). According to comparable trials [28], an effect size of at least d_Cohen_ = 0.33 was assumed, leading to a necessary sample size of 58 NHRs for the primary endpoint.

### Combined intervention

The intervention was developed and executed by the multi-professional project team (nurses, GPs, specialist in internal medicine, clinical pharmacists) by integrating current evidence, expertise and experiences regarding geriatric pharmacotherapy, e-learning and challenges arising during interprofessional cooperation in NHs.

1. A *three-step education* of participating healthcare professionals was conducted (August 2015 – November 2015) addressing medication safety in older adults [7]. The blended-learning concept (combining face-to-face and online training) [29] covered the following topics: drug therapy in older and multi-morbid adults, drug-drug and drug-disease interactions, medication errors, ADE risk groups and monitoring, pharmacovigilance, polypharmacy, potentially inappropriate prescriptions (PIP), over- and under-prescribing, prioritizing and deprescribing of drugs (evaluation of patient’s drug regimens according to current diagnoses and therapy goals and subsequent dose reduction or stopping of medications with lack of benefit or causing potential harm), medication review, legal aspects of drug therapy, strategies to enhance interprofessional cooperation [7].

The training consisted of:

- a kick-off interprofessional face-to-face workshop (3 h)
- three profession-specific online sessions (each 20–45 min) with audio-visual presentations and autonomous processing of case files addressing medication-related problems
- a final interprofessional face-to-face event with discussion of the cases and instructions for the second part of the intervention (1.5 h).

(2) Upon conclusion of the training, the *therapy check process* was conducted (January 2016 – September 2016) with the aim of structuring interprofessional communication within the medication process. An electronic communication tool, the InTherAKT-online Platform (I-oP), was designed for this purpose by the software-engineering company smart-Q (Germany) in collaboration with the project team. The tool was accessible online (https://www.intherakt.de/app) via double-secured access keys. Each participant was granted access solely to the respective residents in treatment.

**Fig. 1:**
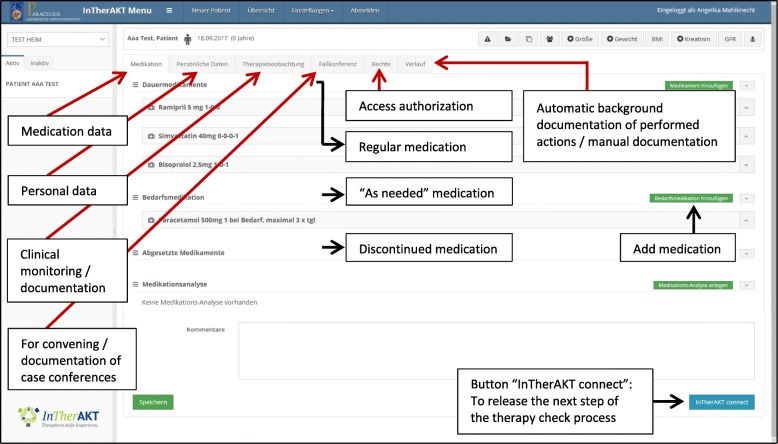
InTherAKT-online Platform (screenshot): surface and medication plan

The system prompted users to enter patient-related data (age, gender, corresponding NH/GP/pharmacist, diagnoses, allergies/drug intolerances, height/weight, creatinine, other relevant laboratory values, physician visits/hospitalizations) and medication-related data (brand name, International Nonproprietary Name, indication, dosage, form/time of administration, indications for administration/monitoring, regular/as-needed medication, prescriber, date of first prescription). The medication plan was based on the German Medication Plan 2.0 [30] (standardized, nationally introduced schedule to improve patient safety by enhancing the information flow between concerning healthcare professionals). Furthermore, the I-oP allowed documentation of patients’ symptoms and to convene and document case conferences (Fig. 1).

**Fig. 2:**
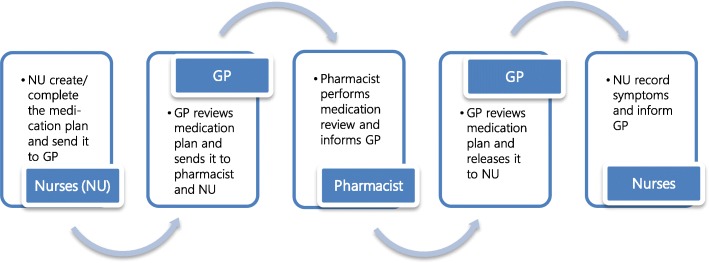
Stages of the therapy check process (NU = nurses, GP = general practitioner)

**Fig. 3:**
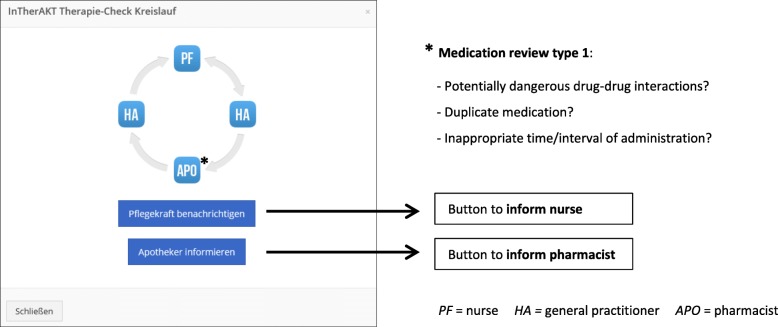
InTherAKT-online Platform (screenshot, logged in as GP) Window displayed after clicking the button “InTherAKT connect” in the medication section → GP is prompted to send a message to nurses and/or pharmacist.

After an intensive testing and revising phase, the tool was authorized for use in the *therapy check process*, which consisted of the following steps (Fig. 2):

- Nurses/study assistants (see below) entered data in the I-oP and informed the responsible GP
- The GP completed the medication plan and performed a comprehensive medical review of the drug regimens based on updated and complete drug/patient information (clinical and laboratory parameters) and on the knowledge acquired during the training. The GP released the reviewed medication plan to the responsible nurses and pharmacist. GPs also had the possibility to ask the pharmacists specific questions relating to medication use.
- The pharmacist performed a medication review type 1 [31] (Fig. 3), informed the GP about suggestions for improvement if applicable
- GP reviewed pharmacist’s suggestions, released the revised medication plan to the nurses and communicated with the NHR and/or legal representative verbally during onsite visits in the NH
- Nurses performed clinical monitoring and weekly documentation of residents’ symptoms (section “Therapiebeobachtung”/therapy monitoring, Fig. 1) [32] and informed the GP in case of unexpected changes
- On demand: organization of multi-disciplinary case conferences if interprofessional decision-making was required.

Communication between healthcare professionals was conducted via a standardized messaging system. Messages were generated automatically whenever the next therapy check step was released and were sent to the email address of the relevant contact person. Due to data security requirements messages contained no patient data, but included a link to the start page of the I-oP (“To-Do” section), accessible via the individual access keys, where all current actions to perform were listed.

Participants used the platform whenever they were advised there was a step to perform and additionally for inputting documentation. In case of an urgent need for action (e.g. acute or severe symptoms), healthcare professionals communicated by phone and retrospectively documented events, decisions and therapeutic implications in the online platform.

**Fig. 4:**
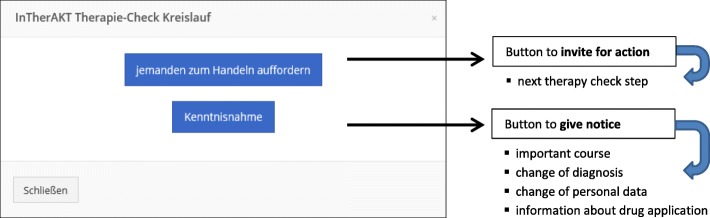
InTherAKT-online Platform (screenshot): two different ways of communication

All participants received profession-specific instruction manuals. After the first intervention period, some functions were revised and optimized, e.g., when clicking “InTherAKT connect”, a dichotomization between “invite for action” and “notice” was introduced (Fig. 4).

Documentation in the I-oP had to be conducted in addition to routine documentation in the respective electronic health records (EHRs) as the tool has not yet been embedded into primary documentation. Thus, study assistants were recruited to aid nurses entering data and to support the therapy check in the NHs in close collaboration with the nurses. The project team supervised all participants; in the second intervention period (between data collection t_1_ and t_2_, see below) this supervision was restricted to technical support and the project team intervened only if activities in the I-oP had ceased for 1 week. Study assistants were supervised continuously during the whole study period by the project team.

The therapy check procedure was planned to be completed at least once for every resident until t_1_ and was intended to re-start at any relevant change of the medication (e.g. new prescription, modification of dosage etc.).

### Outcome measurements

*Primary endpoint*: Appropriateness of prescribed medications, measured via the Medication Appropriateness Index (MAI) [33]. MAI ratings were conducted by two different clinical pharmacists (one at t_0_, one at t_1_/t_2_) who were not study participants. The pharmacists who conducted the MAI ratings were instructed and supervised by the same expert clinical pharmacist throughout the study period. They followed an instruction regarding the use of the MAI which was provided on request by the originators of the MAI (unpublished document, last updated on 2014 Nov 01).

Data used for the MAI rating were collected from the NH documentation (complete list of medications with brand names and International Nonproprietary Names of drugs, indication, dosage, mode of application, duration of therapy, instructions for application; patient-related data: height, weight, age, current ICD-10 coded diagnoses, laboratory values e.g. creatinine, potassium, blood sugar; known drug intolerances, allergies).

The MAI consists of 10 items which are assessed for each prescribed drug. Each item is rated as “appropriate” (0 points) or “inappropriate”; in the latter case, a weighted score is assigned [34] (Table 1). MAI scores per drug are summated to get the sum-score (MAI-Sum) per NHR [28]. Higher sum-scores indicate higher inappropriateness; however, the absolute MAI-Sum is not a direct parameter for medication appropriateness because it depends on the number of drugs. Thus, MAI-Sum shows high variability and therefore we calculated also a MAI-Mean. Mean difference of MAI-Sum (mean MAI change) is used [28] to interpret changes within a sample and to compare results between different studies.

**Table 1:** Primary and secondary outcomes

Primary outcome: Medication Appropriateness Index (MAI)		
Number	Item	Weighted score [34]
1	Is there an indication for the drug?	3
2	Is the medication effective for the condition?	3
3	Is the dosage correct?	2
4	Are the directions correct?	2
5	Are the directions practical?	2
6	Are there clinically significant drug-drug interactions?	2
7	Are there clinically significant drug-disease/condition interactions?	1
8	Is there unnecessary duplication with other drugs?	1
9	Is the duration of the therapy acceptable?	1
10	Is this drug the least expensive alternative compared to others of equal utility?	1
Range of the MAI sum-score per drug: 0 (fully appropriate) – 18 (fully inappropriate)		
MAI-Sum per NHR (dependent on the number of drugs): summation of the weighted MAI sum-scores per drug		
MAI-Mean (independent on the number of drugs): division of MAI-Sum by the number of drugs; range 0–18		
Mean difference (“mean MAI change”): mean difference [28] of MAI-Sum between times of measurement		
Secondary outcomes		
Outcome	Measuring method	Description of measuring method
Cognitive performance	Mini Mental State Examination (MMSE) [35] Dementia Screening Scale (DSS) [36]	Direct testing of patients; < 25 points → cognitive impairment (range 0–30) Proxy-tool, answering of questions related to the patient by nurses; ≥3 points → cognitive impairment (range 0–14)
Clinical signs of delirium	Delirium Observation Screening Scale (DOS) [37]	Proxy-tool, screening instrument for identifying risk patients; ≥3 points → delirium presumable (range 0–13)
Agitation if MMSE < 18 (moderate - severe cognitive impairment) [38]	Cohen Mansfield Agitation Inventory (CMAI-D) [39]	Proxy-tool, related to resident’s behavior in last 2 weeks; four dimensions (physically/verbally aggressive, physically/verbally not aggressive) plus additional item apathy
Mobility/tendency of falls	Timed Get Up and Go test (TUG) [40]	Walking test of patients; ≥10 s. → impaired mobility
Number of drugs	Electronic health records of NHs	Total number of prescribed drugs
Number of severe DDIs	UpToDate [41]	Severe DDIs = category X
Appropriateness of recorded analgesics	Medication Appropriateness Index (MAI) [33]	MAI-Sum and MAI-Mean were calculated as for the primary endpoint but only including prescribed analgesics (ATC classes M01, N02A, N02B)

*DDIs* Drug-drug interactions, *NHs* Nursing homes

*Secondary endpoints* are listed in Table 1.

### Data collection

*Outcome parameters* were collected before (t_0,_ July – October 2015), during (t_1_, May – July 2016) and after the intervention (t_2_, October – December 2016). Due to delays in the recruitment phase (caused by summer vacations and absence of participants), baseline data collection was concluded 2 months later than scheduled and t_0_ concerning 17 NHRs was completed after the first training session. Data collections were performed by trained study assistants in direct contact with NHRs (e.g. MMSE), as proxy-tools with nurses (e.g. DSS) or using the EHR (e.g. medication-related data). Residents’ diagnoses derived from EHRs were compared with the diagnoses registered in the I-oP (filled in by nurses and GPs) and completed when required. After pseudonymization by the study assistants, data were exported for analysis.

Furthermore, *structural data* of participating NHs, pharmacies and GP offices (e.g. number of residents/employees, monthly NH visits per GP) and *variables describing the intervention period* were collected (level of care, number of hospitalizations/physician consultations, falls, performed case conferences, mortality, physical function via Barthel Scale [42], presence of pain via Verbal Rating Scale (VRS) [43] and Pain Assessment in Advanced Dementia Scale (PAINAD) [44]).

### Data analysis

Statistical analysis was performed via IBM®SPSS Statistics®23 using descriptive methods, calculations of effect sizes and Pearson’s correlations. For the primary endpoint, change between t_0_ and t_2_ was analyzed using one-sided T-tests for dependent samples (95%CI, significance level: 0.05). Due to the study’s descriptive nature hypothesis-testing procedures aimed primarily to generate hypotheses.

Based on a recent RCT determining 24 MAI-Sum points as potential cut-off to initiate a medication review [45], NHRs were dichotomized according to their baseline sum-score (< 24/≥24 points). MAI scores were calculated for all NHRs and for these two subgroups.

## Results

### Study participants

**Fig. 5:**
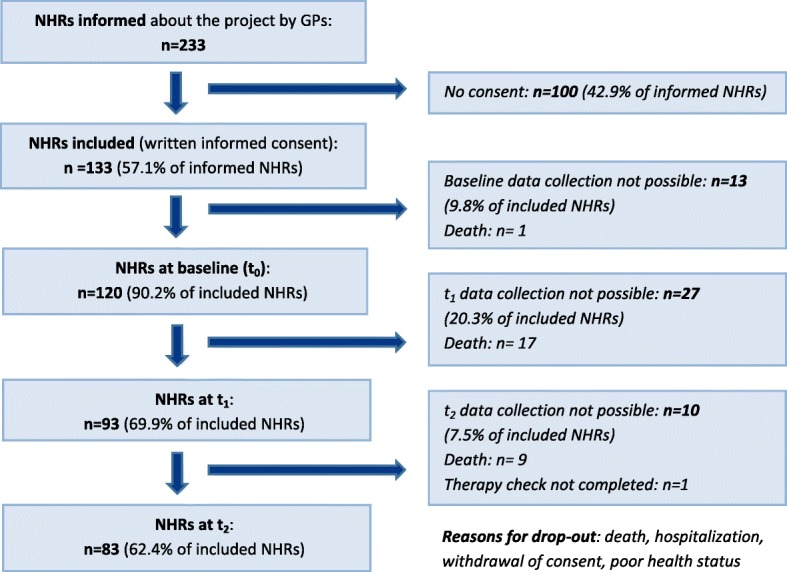
Recruitment and participation of nursing home residents (NHRs)

Fourteen of 120 invited GPs, 9 of 15 invited NHs, 11 of 12 invited pharmacists and 120 of 233 invited NHRs (Fig. 5, Table 2) participated.

**Table 2:** Characteristics of the study participants

Nursing homes: baseline characteristics (*n* = 9)			
Number of care places n	occupied/ total		964/979
Number of employees n	full-time equivalent/headcount		365/578
General practice care M ± SD	collaborating GPs per NH		15.0 ± 6.3
	monthly GP visits per NH (estimated)		3.0 ± 2.2
Pharmaceutical care n	pharmacies supplying the participating NHs		9
Pharmacies: baseline characteristics (*n* = 9) ^a^			
Number of employees M ± SD	state-approved pharmacists (headcount)		3.3 ± 1.3
*GP offices: baseline characteristics (n = 11)* ^a^			
Number of employees M ± SD	GPs (headcount)		2.7 ± 1.3
Number of treated patients M ± SD	estimated count of patients per quarter year		1945 ± 995
Number of treated NHRs M ± SD	estimated count of currently treated NHRs		59 ± 38
NH visits per GP n	not regularly		1
	1/month		4
	2–3/month		5
	> 3/month		1
Residents: baseline characteristics (*n* = 120)			
Age in years M ± SD			85.2 ± 7.0
	Min - max ^b^		62–100
Gender %	Male / female		27.5 / 72.5
Reported diagnoses %	Arterial hypertension		71.0
	Dementia		52.0
	Depression		38.0
	Cerebrovascular disease		34.0
	Cardiac arrhythmias		34.0
	Chronic renal disease		29.0
	Diabetes mellitus		23.0
	Coronary heart disease		22.0
	Chronic heart failure		22.0
	Malignant tumors		15.0
	Chronic obstructive pulmonary disease (COPD)		15.0
Residents: changes of functional characteristics during the study period (*n* = 83) ^c^		t_0_	t_2_
Barthel index range 0 (fully dependent) - 100 (fully independent) M ± SD		51.3 ± 28.7	43.6 ± 28.5
Number of diagnoses per NHR M ± SD		12.3 ± 5.3	14.2 ± 6.9
Presence of pain % (*n* = 71)	self-reported (VRS)/ external assessment (PAINAD)	77.5	66.2
Levels of care n (%)	Care level 0	5 (6%)	3 (3.6%)
	Care level 1	37 (44.6%)	32 (38.6%)
	Care level 2	26 (31.3%)	30 (36.1%)
	Care level 3	14 (16.9%)	17 (20.5%)
	Hardship case	1 (1.2%)	1 (1.2%)
Residents: variables describing the intervention phases		t_0_ – t_1_	t_1_ – t_2_
Physician/hospital consultations (*n* = 83)	number of NHRs n (%)	17 (20.5%)	n.a.
	total number of consultations n	54	n.a.
Falls (*n* = 83)	number of NHRs n (%)	12 (14.5%)	n.a.
	total number of falls n	30	n.a.
Mortality (*n* = 120)	number of deceased NHRs n (%)	17 (14.2%)	9 (7.5%)
Mortality rate per month (*n* = 120)	number of deceased NHRs per 1000 per month n	14	15

*M* Mean, *SD* Standard deviation, *NH* Nursing home, *NHR* Nursing home resident, *VRS* Verbal Rating Scale, *PAINAD* Pain Assessment in Advanced Dementia Scale, *n.a.* not available

^a^ Structural data were not available from 2 pharmacies and 3 GP offices

^b^ Two residents were under 65 years, yet included in the analysis

^c^ Residents with data collected at all testing times

83 NHRs completed the intervention. No difference was found between drop-outs and remaining NHRs regarding MAI-sum (T_df = 118_ = 0.83, *p* = .409) or MAI-mean (T118_df = 118_ = 0.98, *p* = .329) at baseline

### Intervention

All institutions were represented at the first onsite training with the exception of one GP. As the first training session was recorded, footage of this event could be provided to all participants.

To ensure that participants could watch *all* profession-specific online presentations, access keys were not personalized; thus, distinct individualized assignment of accesses was not available.

Participants not attending the final event (6 GPs, 3 pharmacists, 3 NHs) were instructed personally about the therapy check.

At t_1_, one full therapy check was completed for 35 NHRs (42.2%). 15 (18.1%) of the cycles were at the point where the first GP’s review had been sent to the pharmacist, 30 (36.1%) where at the point after the conduction of the pharmacist’s review (Fig. 2).

At t_2_, at least one therapy check was completed for all NHRs except for one who was consequently excluded from analysis. A second full cycle was completed in 7 cases (8.4%). For further 9 NHRs (10.8%) a second pharmacist’s review was performed without completing the cycle. Communication steps without medication review were performed for further 15 NHRs (18.1%).

No case conferences took place.

### Primary endpoint

**Fig. 6:**
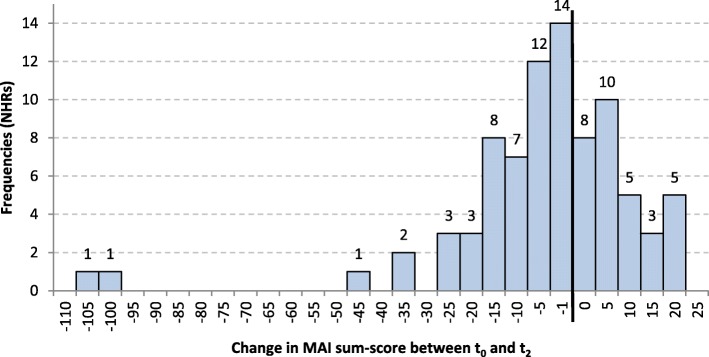
Distribution of change in MAI-Sum between t_0_ and t_2_ (*n* = 83)

Sixty-two point seven percent of NHRs showed a reduction in MAI-Score over time. Distribution of MAI change is shown in Fig. 6. MAI-Sum and MAI-Mean decreased significantly. Change in MAI-Sum showed a small effect (d = 0.39) with a mean reduction of 7.1 ± 19.6 and a median reduction of 3.0 between t_0_ and t_2_. As mean variation of MAI-Sum was high due to different numbers of drugs, change in MAI-Mean showed a higher effect (d = 0.61).

Mean MAI change and effect size were high for NHRs with baseline MAI-Sum ≥24 and showed no effect for those with MAI-Sum < 24. A strong association between baseline MAI-Sum and mean MAI change was found: the higher MAI-Sum at baseline, the higher the mean change (thus, the stronger the improvement). MAI-Sum at baseline explained 32.8% of the variance of MAI change (Table 3).

**Table 3:** Results: primary outcome

Primary endpoint: Medication Appropriateness Index (MAI)								
* n* = 83						Mean difference (MAI change) [CI_95%_]	Effect size d_cohen_ [CI_95%_]	*p*-value (1-tailed)
			t_0_	t_1_	t_2_	t_2_- t_0_	t_2_- t_0_	t_2_- t_0_
MAI-Sum		M ± SD	23.2 ± 22.5	18.3 ± 13.1	16.1 ± 12.7	-7.1 [−11.4 – −2.8]	−0.39 [− 0.82–0.05]	0.001
		MD (IQR)	18.0 (9.0–32.0)	16.0 (9.0–27.1)	13.0 (7.0–26.0)	−3.0 (− 13.0–2.0)		
Subgroup	MAI-Sum ≥24 at t_0_ (*n* = 31)	M ± SD	43.2 ± 25.5	29.8 ± 11.7	25.8 ± 12.9	−17.4 [−27.6 **–** −7.2]	−0.86 [− 1.60 **–** − 0.13]	0.001
		MD (IQR)	36.0 (30.0–45.0)	30.0 (19.0–36.0)	26.0 (14.0–34.0)	− 15.0 (− 25.0 – − 2.0)		
	MAI-Sum < 24 at t_0_ (*n* = 52)	M ± SD	11.2 ± 6.7	11.4 ± 8.3	10.3 ± 8.4	−0.9 [−3.2–1.3]	−0.12 [− 0.67–0.42]	0.197
		MD (IQR)	11.0 (7.0–16.8)	11.0 (4.5–17.0)	9.0 (5.0–14.0)	− 1.0 (− 5.0–2.0)		
MAI-Mean		M ± SD	1.9 ± 1.2	1.5 ± 0.9	1.3 ± 0.8	−0.6 [− 0.8 – − 0.4]	−0.61 [− 1.05 **–** − 0.17]	0.000
		MD (IQR)	1.8 (1.2–2.6)	1.5 (1.0–2.0)	1.3 (0.8–1.8)	− 0.3 (− 1.3–0.1)		
Pearson correlation between MAI sum-score at t0 and mean difference t2- t0*: r = −* 0.83								
* n* = 81 ^a^						Mean difference (MAI change) [CI_95%_]	Effect size d_cohen_ [CI_95%_]	*p*-value (1-tailed)
			t_0_	t_1_	t_2_	t_2_- t_0_	t_2_- t_0_	t_2_- t_0_
MAI-Sum		M ± SD	20.5 ± 14.6	17.9 ± 13.0	15.8 ± 12.7	−4.7 [−7.5 **–** −1.9]	−0.35 [− 0.78–0.09]	0.001
		MD (IQR)	17.0 (9.0–30.5)	16.0 (8.5–26.0)	12.0 (7.0–22.5)	− 3.0 (− 12.0–2.0)		
Subgroup	MAI-Sum ≥24 at t_0_ (*n* = 29)	M ± SD	37.1 ± 9.2	29.4 ± 12.0	25.6 ± 13.3	−11.5 [− 17.8 – −5.3]	−1.00 [− 1.77 **–** − 0.23]	0.000
		MD (IQR)	34.0 (29.5–42.0)	29.0 (19.0–36.0)	26.0 (14.0–34.0)	− 14.0 (− 22.0 – − 0.5)		
	MAI-Sum < 24 at t_0_ (*n* = 52)	M ± SD	11.2 ± 6.7	11.4 ± 8.3	10.3 ± 8.4	−0.9 [−3.2–1.3]	−0.12 [− 0.67–0.42]	0.197
		MD (IQR)	11.0 (7.0–16.8)	11.0 (4.5–17.0)	9.0 (5.0–14.0)	− 1.0 (− 5.0–2.0)		
*Linear regression to predict the mean difference t*_*2*_ *- t*_*0*_ *(MAI-change) by MAI-Sum at t*_*0*_ *:* *ß = − 0.57 (p = .000), R*^*2*^ *= 0.33 (p = 0.000)*								

*M* Mean, *SD* Standard deviation, *MD* Median, *IQR* Inter-Quartile-Range, *MAI-Sum* Weighted MAI sum-score, *MAI-Mean* Weighted MAI mean-score

^a^ To additionally depict the mean more adequately, two outliers with high baseline sum-scores/change values > 4 SD greater than the mean and high baseline numbers of drugs/marked drops in drug count were excluded

Table 4 shows detailed descriptive results of MAI-Sum and mean MAI change by the single MAI items for the whole sample (*n* = 83). The greatest mean MAI changes of > 1 point were noted in item 1 (indication), item 10 (costs) and item 3 (dosage). The MAI-Sum of item 6 (clinically significant drug-drug interactions) increased over time. After exclusion of two outliers with extraordinary high baseline sum-scores (Table 3), results did not change substantially. The subgroup with MAI-Sum ≥24 at baseline showed stronger MAI changes in all items while MAI changes were small in the subgroup with MAI-Sum < 24.

**Table 4:** Weighted MAI sum-score by MAI items

Primary endpoint: Descriptive results by MAI items					
*n* = 83		MAI-Sum M ± SD			Mean difference
Nr.	Item	t_0_	t_1_	t_2_	t_2_- t_0_
1	Is there an indication for the drug?	7.2 ± 8.6	5.1 ± 5.0	5.2 ± 5.5	−2.0
2	Is the medication effective for the condition?	1.1 ± 2.2	0.4 ± 1.1	0.3 ± 1.0	−0.8
3	Is the dosage correct?	2.2 ± 3.0	1.5 ± 2.1	1.1 ± 1.5	−1.1
4	Are the directions correct?	3.1 ± 3.5	2.9 ± 2.8	2.2 ± 2.6	−0.9
5	Are the directions practical?	0.5 ± 1.4	0.0 ± 0.0	0.0 ± 0.2	−0.4
6	Are there clinically significant drug-drug interactions?	1.7 ± 2.8	2.7 ± 3.6	2.7 ± 3.8	1.0
7	Are there clinically significant drug-disease/condition interactions?	0.3 ± 0.7	0.2 ± 0.5	0.1 ± 0.3	−0.3
8	Is there unnecessary duplication with other drugs?	0.4 ± 0.8	0.3 ± 1.1	0.2 ± 0.6	−0.2
9	Is the duration of the therapy acceptable?	2.7 ± 2.9	1.9 ± 1.8	1.8 ± 1.9	−0.8
10	Is this drug the least expensive alternative compared to others of equal utility?	4.1 ± 3.7	3.2 ± 2.5	2.5 ± 2.0	−1.6

*MAI-Sum* Weighted MAI sum-score, *M* Mean, *SD* Standard deviation

Table 5 shows detailed descriptive results of MAI-Sum by drug classes according to ATC level 2.

**Table 5:** Weighted MAI sum-score by drug classes

Primary endpoint: Descriptive results by ATC-class level 2				
*n* = 83	MAI-Sum M ± SD (n)			Mean difference
ATC-class level 2 ^a^	t_0_	t_1_	t_2_	t_2_- t_0_
A02 – Drugs for acid related disorders	5.4 ± 3.8 (44)	3.4 ± 2.8 (45)	2.7 ± 2.5 (44)	−2.7 ^b^
A06 – Laxatives	1.1 ± 2.1 (74)	0.4 ± 1.0 (79)	0.4 ± 0.9 (87)	−0.7
A07 – Antidiarrheals, intestinal antiinflammatory/antiinfective agents	1.5 ± 2.8 (15)	0.9 ± 1.8 (15)	0.5 ± 1.5 (11)	−1.0 ^b^
A10 – Drugs used in diabetes	2.3 ± 3.3 (23)	2.6 ± 3.6 (27)	0.9 ± 1.9 (24)	−1.4 ^b^
A11 – Vitamins	3.3 ± 2.5 (24)	2.4 ± 2.8 (31)	2.7 ± 3.2 (36)	−0.6
B01 – Antithrombotic agents	1.4 ± 2.4 (54)	1.1 ± 2.0 (56)	0.9 ± 2.0 (57)	−0.5
B03 – Antianemic preparations	2.2 ± 2.9 (13)	1.9 ± 2.6 (17)	2.2 ± 4.5 (19)	0.0
C01 – Cardiac therapy	3.1 ± 2.5 (18)	1.3 ± 1.9 (18)	0.5 ± 0.9 (17)	−2.5 ^b^
C03 – Diuretics	1.7 ± 2.3 (51)	1.5 ± 2.2 (52)	1.4 ± 2.4 (51)	−0.3
C07 – Beta blocking agents	1.2 ± 1.8 (40)	1.5 ± 1.8 (37)	1.0 ± 1.5 (37)	−0.2
C08 – Calcium channel blockers	2.0 ± 2.7 (23)	1.5 ± 2.3 (23)	1.2 ± 1.7 (24)	−0.8
C09 – Agents acting on the renin-angiotensin system	0.9 ± 1.2 (40)	0.8 ± 1.2 (40)	0.5 ± 1.1 (40)	−0.4
C10 – Lipid modifying agents	1.1 ± 1.8 (25)	0.7 ± 1.4 (22)	0.5 ± 1.3 (22)	−0.6
D01 – Antifungals for dermatological use	5.7 ± 6.0 (19)	3.8 ± 4.4 (13)	2.4 ± 3.9 (10)	−3.3 ^b^
D07 – Corticosteroids, dermatological preparations	4.6 ± 4.1 (14)	4.8 ± 2.9 (14)	3.9 ± 3.4 (12)	−0.7
G04 – Urologicals	2.0 ± 2.7 (12)	2.2 ± 1.9 (9)	2.0 ± 2.5 (8)	0.0
H03 – Thyroid therapy	2.8 ± 2.7 (22)	2.5 ± 2.5 (22)	2.4 ± 3.0 (22)	−0.4
M01 – Antiinflammatory and antirheumatic products	3.2 ± 3.3 (13)	1.0 ± 1.2 (11)	1.7 ± 2.9 (7)	−1.5^b^
N02 – Analgesics	1.5 ± 2.5 (94)	1.1 ± 1.7 (109)	0.8 ± 1.3 (99)	−0.7
N03 – Antiepileptics	1.1 ± 1.6 (20)	2.2 ± 2.2 (19)	2.4 ± 2.6 (21)	1.2 ^c^
N04 – Anti-Parkinson drugs	3.4 ± 2.3 (21)	5.4 ± 4.8 (27)	3.6 ± 4.8 (27)	0.3
N05 – Psycholeptics	6.0 ± 5.7 (80)	4.4 ± 4.7 (89)	3.8 ± 4.7 (84)	−2.2 ^b^
N06 – Psychoanaleptics	2.1 ± 2.2 (50)	2.8 ± 2.4 (43)	2.6 ± 2.5 (48)	0.5
R03 – Anti-asthmatics	7.8 ± 13.4 (26)	3.4 ± 3.6 (29)	2.7 ± 4.7 (26)	−5.1 ^b^
S01 – Ophthalmologicals	4.9 ± 6.3 (27)	3.5 ± 2.9 (18)	3.0 ± 3.8 (22)	−1.9 ^b^

*MAI-Sum* Weighted MAI sum-score, *M* Mean, *SD* Standard deviation, *(n)* frequency of prescription

^a^ Only drug classes represented by > 10 prescriptions at baseline were considered

^b^ Strongest decreases of weighted MAI sum-score: mean difference ≤ − 1

^c^ Strongest increases of weighted MAI sum-score: mean difference ≥ + 1

The strongest decreases of MAI-Sum were found for anti-asthmatics, antifungals for dermatological use, drugs for acid-related disorders, cardiac therapy (glycosides, antiarrhythmics, cardiac stimulants, vasodilators) and psycholeptics (antipsychotics, anxiolytics, hypnotics and sedatives). Despite of the notable decreases of MAI-Sum, in most of these drug classes, the number of prescriptions did not change substantially over time. An exception was the use of antifungals for dermatological use which dropped nearly by half.

Analgesics and psycholeptics were the most frequently prescribed drug classes. Other commonly used drug classes were: laxatives, antithrombotic agents, diuretics, psychoanaleptics (antidepressants, psychostimulants, anti-dementia drugs, nootropics etc.) and drugs for acid-related disorders. All these frequently used drug classes showed a reduction of MAI-Sum except psychoanaleptics where MAI-Sum slightly increased. An increase of MAI-Sum was noted also regarding antiepileptics.

### Secondary endpoints

At t_0_, 67.9% of NHRs were cognitively impaired (45.7% moderately or severely). Mean MMSE-score decreased slightly over time but showing no relevant effect. The DSS-score showed a small effect for declining cognitive function.

All NHRs had impaired mobility, 50% were unable to walk. TUG-time remained constant over time. Nearly 60% displayed signs of delirium. Percentage of cognitively impaired NHRs with agitated behavior decreased between t_1_ and t_2_, apathetic behavior increased.

Polypharmacy was frequent and increased slightly over time, number of drugs remained constant as did frequency of hyper-polypharmacy (≥10 prescriptions). Mean number of severe DDIs increased slightly. 52 NHRs (62.7%) were treated with analgesics throughout the duration of the study period. MAI scores of analgesics decreased over time (Table 6).

**Table 6:** Results: secondary outcomes

Secondary endpoints					
					Effect size d_cohen_
		t_0_	t_1_	t_2_	t_2_- t_0_
MMSE-score M ± SD (*n* = 81)		17.4 ± 9.3	16.4 ± 10.0	16.6 ± 9.6	0.08
DSS-score M ± SD (*n* = 80)		4.7 ± 4.3	5.2 ± 4.3	5.6 ± 4.8	0.19
TUG-time (sec) M ± SD (*n* = 34)		30.3 ± 16.9	32.3 ± 19.8	29.5 ± 12.9	0.07
CMAI-D signs of agitation % (*n* = 31)		83.9	83.9	67.8	
Apathy %		25.8	29.0	38.7	
DOS signs of delirium % (*n* = 62)		58.1	62.9	59.7	
Number of drugs per NHR M ± SD (*n* = 83)		11.0 ± 5.3	11.5 ± 5.1	11.3 ± 4.7	0.06
Min - Max		1–29	3–28	4–24	
Regular prescriptions per NHR M ± SD		8.2 ± 4.2	8.5 ± 4.1	8.5 ± 4.1	0.07
“As needed” prescriptions per NHR M ± SD		2.7 ± 2.0	3.1 ± 1.9	2.8 ± 1.6	0.04
NHRs with ≥5 drugs %		79.5	80.7	83.1	
NHRs with ≥10 drugs %		37.4	37.4	37.4	
S-DDIs per NHR M ± SD (*n* = 83)		0.18 ± 0.47	0.19 ± 0.50	0.27 ± 0.60	0.17
% NHRs with ≥1 S-DDIs		14.4	14.4	20.5	
Analgesics MAI-Sum (*n* = 52) (ATC classes M01, N02A, N02B)	M ± SD	2.4 ± 3.4	1.3 ± 1.9	1.2 ± 1.8	0.45
	MD (IQR)	1.0 (0.0–3.8)	0.0 (0.0–2.0)	0.0 (0.0–2.0)	
Analgesics MAI-Mean (*n* = 52) (ATC classes M01, N02A, N02B)	M ± SD	1.3 ± 2.0	0.6 ± 0.8	0.6 ± 0.9	0.46
	MD (IQR)	0.5 (0.0–2.0)	0.0 (0.0–1.0)	0.0 (0.0–.90)	

*MMSE* Mini Mental State Examination, *DSS* Dementia Screening Scale, *TUG* Timed Get Up and Go Test, *NHR* Nursing home resident, *S-DDIs S*evere drug-drug interactions

## Discussion

### Summary and interpretation of findings

Investigated NHRs were old-aged and multi-morbid with tendencies to deterioration of physical functions. Medication appropriateness increased significantly during the study period showing a moderate effect similar to previous studies [21, 28]. The effect found for the change in MAI-Sum slightly exceeded the lowest effect size expected in a-priori power analysis. Since pain treatment is common and relevant in NHRs [44] we analyzed the appropriateness of analgesics specifically noting an increase through the study period.

Defined thresholds for clinically relevant MAI changes are not yet in existence [46]. A German RCT (Rose et al.) [45] used a mean reduction of 3.88 found in a Cochrane Review [21] as cut-off-value for a “major benefit” from the intervention. Our findings were above this threshold, the median was beneath. As mean variation of MAI-Sum was high, the median seems to be a more adequate measure of MAI change. Congruently to the findings of Rose et al. [45], our subgroup analysis demonstrated that NHRs with high baseline MAI-Sum derived a “major benefit” from the intervention in terms of enhanced medication appropriateness, while lower baseline scores barely left room for improvement. However, it remains unclear which extent of improved medication appropriateness results in *clinically* significant benefits for patients.

The strongest improvements were shown regarding the MAI items indication and costs. This could be related to potential educational effects of the training on prescribers and to the fact that the lists of diagnoses and medication plans were revised and updated during the therapy check. Thus, the improvements regarding indication may reflect a more complete documentation of diagnoses.

The item drug-disease interaction was scored relatively low in our sample. The inconsistent documentation of falls throughout the study period (Table 2) may have contributed to this finding in terms of low detection of a risk condition for drug-disease interactions.

Patient-related outcomes did not change substantially aside from a decreased level of agitation. Similarly, current systematic reviews assessing comparable interventions and settings demonstrated improvements of medication appropriateness, but no significant changes of mortality, hospitalization or quality of life [21, 47]. Though, improvements are probably difficult to achieve given the physio-pathologic preconditions in NHRs. An epidemiologic study [48] described a mean cognitive decline of 0.6 MMSE-points/year in populations without dementia and higher declines (2.3/year) in demented persons. In our sample, a decline of 0.6/year was found in a mixed population (45.7% had moderate or severe cognitive impairments). Another study [49] showed a mean decline of 0.5–0.9/year; baseline MMSE-mean in this population was higher than in our study (24.5 vs. 17.4). Thus, cognitive decline in our sample may cautiously be interpreted as positively affected in terms of deceleration compared to epidemiologic data. Frequency of apathetic behavior increased during the study period which could be related to physical deterioration.

Although medication appropriateness increased, mean number of drugs was high and did not change substantially. This phenomenon was also found in other research [50]. There might have been missed opportunities to deprescribing for different reasons, e.g. GPs not identifying overtreatment or low perceived self-efficacy to deprescribe. However, the MAI items indication and duration of drug therapy showed improvements despite of the high number of drugs; moreover, the analysis according to ATC-classes showed that medication appropriateness improved in nearly all of the most frequently used drug classes, and that the drug classes with the highest improvements in medication appropriateness did not show substantial variations regarding numbers of prescriptions. This may underpin the hypothesis that polymedication is not necessarily synonymous with inappropriateness [51, 52].

Despite enhanced medication appropriateness and the intervention with medication review and DDI check, mean number of severe DDIs increased. Previous studies found similar results [53, 54]. This may be related to multi-morbidity and polypharmacy which are associated with an elevated risk of DDIs [52, 55]. In daily routine, if the expected benefit outweighs the risk, DDIs are sometimes accepted under appropriate patient monitoring [56].

Mortality rates in our sample were similar to observational [49] and other interventional NH studies [10, 22, 23, 25] and correspond to presupposed mortality in this population due to age and morbidity.

### Strengths and limitations

A strength is the innovative and multi-faceted approach [57] combining blended-learning education and HIT-based structuring of interprofessional communication. As previously described, implementation of HIT requires considering socio-organizational factors [58] besides providing technical preconditions. We addressed this by involving participants in the design process and by adapting the online platform to suit the participants’ organizational settings. As verbally reported by the study participants, onsite trainings had positive effects on the social relationships between healthcare professionals (details of qualitative results will be published separately). Herewith, communication was not only standardized, but also individualized [59].

The online platform enabled healthcare professionals to perform the therapy check flexibly and independently of time and location which was well accepted. This can be considered an advantage especially for the NH setting with geographically dispersed players [60] and different availabilities.

Another strength is the multi-professional approach involving not only prescribers but all healthcare professionals concerned [47, 61] and their direct interaction [9] based on equal comprehensive and updated patient-related data. There were no case conferences as all concerns and questions raised were resolved during the therapy check (in urgent cases by phone or during routine NH visits).

The primary outcome was investigated using a thoroughly validated implicit instrument [9, 62] involving medication-related and clinical parameters. The MAI, however, does not assess under-prescribing, ADEs and adherence [63].

The study’s main limitation is the non-controlled design which was chosen due to recruitment challenges based on the risk of contamination related to the fact that the study was conducted within one city where GPs treat patients in several different NHs [7]. Another limitation is the application of inference-statistical procedures although participants were not randomly recruited. Hence, results cannot be generalized.

The I-oP was developed and tested within this project. It was not a-priori integrated into the different EHRs used by participants; therefore, records in the platform had to be additionally performed for securing routine documentation. For GPs and pharmacies, this did not restrict the intervention; in the NHs, the therapy check was feasible only by utilizing study assistants and thereby involving nurses in a less direct fashion than planned. This may have caused delays and potential incompletion of the process as specialists’ new prescriptions were not always immediately included in the therapy check or may have been missed when occurring during the ongoing therapy check cycle. These challenges will be addressed by integrating the platform into primary documentation systems.

Even though the study combined medication-related and patient-related outcomes, other relevant endpoints according to the core outcome set for trials of medication review in multi-morbid older patients with polypharmacy were not measured; e.g. drug underuse, drug-related hospital admissions, health-related quality of life [64].

The rather high medication appropriateness could partly be related to selection bias (highly motivated GPs were probably more likely to participate) or study effects (awareness of GPs about surveillance may have influenced their prescribing behavior). The clinical pharmacists conducting MAI ratings were blinded regarding the single patient IDs, but not regarding the different times of measurements. A bias towards a generally more favorable evaluation at the end of the study can, therefore, not be excluded.

Another limitation results from the fact that two different clinical pharmacists conducted the MAI ratings. A calculation of Inter-Rater-Reliability was neither feasible within one testing time (because *one* person conducted the ratings at each time of measurement) nor between the different times of measurement (due to the effect of the intervention between the different testing times that would affect the results). Thus, the possibility of biased results due to inter-rater differences cannot fully be excluded. Nevertheless, both raters were supervised by the same adept clinical pharmacist who checked subsamples of all ratings before approving data for statistical analysis.

### Implications for the future

To facilitate the use and allow optimal alignment with pre-existing workflows [65], the platform is planned to be connected with applied EHRs. Based on the experiences of the supervision process, we recommend thorough training to enable healthcare professionals to perform the therapy check autonomously in the daily routine.

Improvements in medication appropriateness were mainly shown for NHRs with high baseline MAI sum-scores; hence, for future application, the platform could be linked with a CDS counting numbers of drugs and/or based on a list of potentially inappropriate medications (e.g. STOPP/START criteria) [66] which alerts healthcare professionals to initiate a therapy check in cases with high numbers of drugs and/or presence of inappropriate medications.

Providing an app for mobile use could be an attractive solution [67]. In addition, the platform may be adapted for different settings, such as management of chronic diseases in ambulatory care.

## Conclusion

This study was the first to evaluate the impact of a HIT-based intervention addressing communication in the German NH setting. Medication appropriateness increased substantially especially for NHRs with high numbers of drugs and/or inappropriate medication at baseline. Furthermore, some results indicate that there may also be a remote impact on patient-related outcomes in terms of mitigating the natural decline. An appropriate controlled study in course of future application of the communication platform is required to confirm these effects. Future research will be needed to investigate the association between improvement of medication appropriateness and patient-relevant outcomes.

## Data Availability

The datasets used and analyzed during the current study are available from the corresponding author on reasonable request.

## Abbreviations

ADE: Adverse drug event
BESD: Beobachtung von Schmerz bei Demenz, German version of PAINAD
CDS: Clinical decision support
CMAI-D: Cohen Mansfield Agitation Inventory
CPOE: Computerized physician order entry
DDIs: Drug-drug interactions
DOS: Delirium Observation Screening Scale
DRKS: German Clinical Trials Register
DSS: Dementia Screening Scale
GP: General practitioner
EHR(s): Electronic health records
HIT: Health information technology
InTherAKT: “Initiative zur Therapiesicherheit in der Altenhilfe durch Kooperation und Teamwork”- Initiative for medication safety in long-term care via cooperation and teamwork
I-oP: InTherAKT- online Platform
IQR: Inter-Quartile-Range
M: Mean
MD: Median
MAI: Medication Appropriateness Index
MAI-Mean: Weighted MAI mean-score
MAI-Sum: Weighted MAI sum-score
MMSE: Mini Mental State Examination
NH(s): Nursing home(s)
NHR(s): Nursing home resident(s)
PAINAD: Pain Assessment in Advanced Dementia Scale
PIP: Potentially inappropriate prescription
S-DDIs: Severe drug-drug interactions
RCT: Randomized controlled trial
SD: Standard deviation
STOPP/START criteria: Screening Tool of Older People’s Prescriptions/Screening Tool to Alert to Right Treatment
TUG: Timed Get Up and Go test
VRS: Verbal Rating Scale
WHO: World Health Organization
